# Multiresistant bacteria in icu: in the right path

**DOI:** 10.1186/2197-425X-3-S1-A122

**Published:** 2015-10-01

**Authors:** O Moreno, E Yuste, ME Poyatos, M Muñoz, FM Acosta, AM Perez

**Affiliations:** ICU, Hospital Universitario San Cecilio, Granada, Spain

## Introduction

Multiresistant bacteria (MRB) have become a severe public health problem, a risk factor for increasing morbibity, mortality and hospital length of stay and secondarily a raise of social and health resources consumption. The Spanish Health System started in 2014 a national campaign (Zero Resistance) conducted to prevent the emerge of antimicrobial resistance, specially this MRB in intensive care units (ICUs).

## Objectives

To describe the impact of an educational program (Zero Resistance in ICU) on the incidence of MRB in a ICU hospital of Granada, both in infected and colonized patients, dividing into extra and intra ICU nosocomial acquisition. We also describe MRB frequencies and map of resistances.

## Methods

An educational campaign recommended by the Spanish National Health System was driven by the ICU team (physicians, nurses, assistants...) at the end of the first semester 2014. The program consisted in the implementation of 12 preventive measures (table 1). We realized an observational study based on ENVIN-HELICS database of the patients who had MRB positive cultures identifying the microorganisms at admission (nosocomial extra-ICU) and after 48 hours of ICU admission (nosocomial intra-ICU), including infection and colonization, during the year 2014 in our ICU (18 beds). From these results we analyse the frequencies, before (first semester) and after (second semester) implementing the campaign.

## Results

We had a total of 1005 patients in our ICU in 2014: mean age 63.40 years CI95(62.4-64.4), 64.88% male, mean APACHE II 12.84 CI95(12.03-13.62), mean length of stay 5.07 days (SD 8.44). We registered 47 results of MRB cultures: 19 (1.84%) in admission, and 28 (2.71%) after 48 hours in ICU, with a 6.58 ID (for each 1000 days of stay). 26 were infections and 21 colonizations.

From the total (47), during the first six months of 2014 (were the program started) we registered 27 (4.83%): 11 (2%) in admission and 16 (2.97%) after 48 hours, with a ID 6.82; 15 infections, 5 (0.93%) in admission and 10 (1.86%) after 48 hours, with a ID 4.26; and 12 colonizations. In the last semester we registered 20 (4%): 8 (1.61%) in admission and 12 (2.42%) after 48 hours. From these, 11 were infections, 4 (0.81%) in admission and 7 (1.41%) after 48 hours, with a ID of 3.67; and 9 colonizations.

The MRB frequency was (including infection and colonization): MRSA 15.25%, Pseudomona 18.65%, Acinetobacter baumanii imipenem-resistant 35%, Enterobacteria BLEE 6.45%, GNB carbapenemasa 4%.

## Conclusions

The right path it has been achieved as we reached an MRB Incidence of Density reduction of 13.86% since the implementation of the Zero Resistance National Program, even though our incidence of MRB infection and colonization is still above the standard. Most frequent MRB in our environment is A. baumanii, and the less frequent is GNB carbapenemasa.

**Figure 1:**
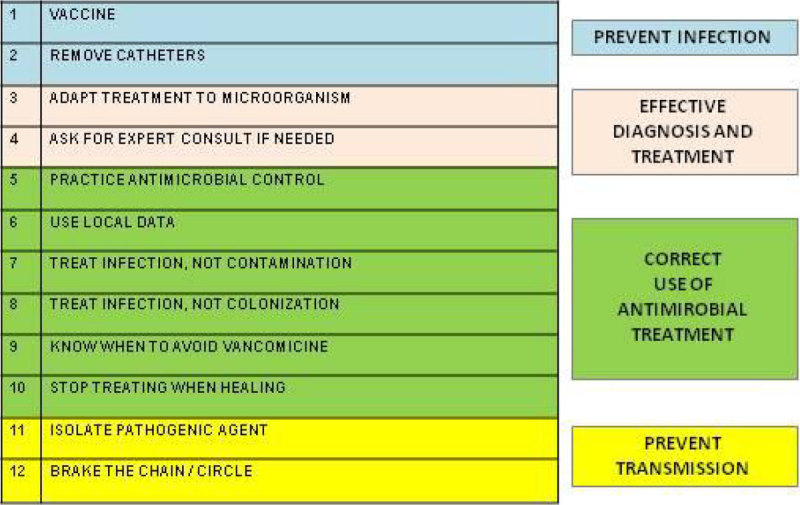
Figure 1

